# Green advertising is more environmentally friendly? The influence of advertising color on consumers’ preferences for green products

**DOI:** 10.3389/fpsyg.2022.959746

**Published:** 2022-10-27

**Authors:** Feng Wenting, Zeng Yuelong, Shen Xianyun, Liu Chenling

**Affiliations:** ^1^Gemmological Institute, China University of Geosciences, Wuhan, Hubei, China; ^2^Research Center for Psychological and Health Sciences, China University of Geosciences, Wuhan, Hubei, China

**Keywords:** green products, advertising color, self-control system, the product type, consumers’ preferences

## Abstract

The color of green product advertisements is an important factor affecting consumers’ preferences. Based on the theory of the self-control system, this paper explores the influence mechanism and boundary conditions of green product ad color on consumers’ preferences through three experiments. Experiment 1 tested the effect of advertisement color type (green/color) on consumers’ preferences for green products. The results show that color ad can promote consumers’ preferences for green products compared with green ad. Experiment 1 also analyzed the mediating role of the self-control system between advertisement color type (green/color) and consumers’ preferences. Experiment 2 further clarified the boundary of the main effect. The effect of ad color (green/color) on consumers’ preferences was only effective in the context of green products. Experiment 3 explored the moderating effect of green product type (egoistic/altruistic) on the main effect. The results show that only when the green product type is altruistic, the ad color type (green/color) can significantly affect consumers’ preferences. This study is the first to link the ad color of green products with consumers’ preferences. The findings confirm that the use of color ad for green products can elicit higher consumers’ preferences than pure green ad, which enriches the research on the color of green product advertisements.

## Introduction

Human activities have led to increasingly serious global environmental problems (Dong et al., 2017), such as the greenhouse effect, air pollution, water shortages, and the extinction of scarce animals (Chen and Lee, 2015; Wang et al., 2016). The aggravation of environmental issues makes people pay more attention to protect the environment(Fu et al., 2022). Incentivizing consumers to engage in green consumption is a very effective marketing strategy (choosing to buy green products instead of traditional products; White et al., 2019; Yan et al., 2019; Tezer and Bodur, 2020). Green products are highly correlated with attributes such as energy saving, environmental protection, and health. Researchers expect long-term environmental benefits from shifting preferences to green products (Sachdeva et al., 2015). However, the market performance of green products is not ideal (Pancer et al., 2017). Consumers’ preferences for green products are not significant. Some consumers stop participating after short-term trials, resulting in insufficient green consumption (Luchs et al., 2010). Therefore, analyzing the relevant factors that affect consumers’ purchase of green products has become a key issue in improving green consumption.

Previous studies have explored the relevant factors that affect consumers’ purchase of green products from multiple perspectives. Consumers’ preferences for green products are influenced by various contextual factors (Meng and Trudel, 2017; Pancer et al., 2017; Seo and Scammon, 2017). Advertising is an important means to encourage consumers to engage in green consumption (Chan, 2000; Goldstein et al., 2008). Advertising that contains environmental claims conveys its message more deeply by discussing the relationship between products and the environment (Leonidou and Hultman, 2011). Existing research has focused on advertising content (Darley and Smith, 1993; Leonidou and Hultman, 2011; White and Simpson, 2013; Green and Peloza, 2014) or presentation (Phillips and Gyoerick, 1999; Miller and Stoica, 2004; Heiser et al., 2008) on consumers’ purchase of green products. Prior studies did not investigate the effect of advertising color type (green/color) on consumers’ preferences for green products. However, in real life, the color types of green product advertisements can be mainly divided into two categories (green/color; Amy, 2013; Jiang et al., 2019). Due to the particularity of green products, some companies use the green color in their advertisements to highlight the green attributes of products (Jiang et al., 2019). For example, Chando’s green cosmetics advertisements use pure green as a whole to show the environmental protection attributes (Lillian, 2020). Starbucks and Whole Foods Market, known for eco-friendliness, signal their sustainability by using a green logo as a particularly strong signal indicating socially desirable business practices (Mazar and Zhong, 2010; Yoon and Oh, 2016). At the same time, color advertisements for green products also exist in the market. For example, the green cosmetics advertisements of Herborist use color ad to display the characteristics of the products themselves (Amy, 2013). The Body Shop, which has been advocating for an end to animal testing since 1989, has long used colorful ad to promote its green products (Lisa, 2022). So, does the color type (green/color) of green product advertisements affects consumers’ preferences? What is the internal mechanism? Existing research is difficult to give insightful answers.

Existing research shows that green products can simultaneously meet the dual goals of assimilation (blending in) and differentiation (maintaining uniqueness). Self-control system theory specifically explains how people deal with multiple simultaneous goals. Therefore, based on the self-control system theory, this study explores the effect of ad color type (green vs. color) on consumers’ preferences for green products. The study consisted of three experiments in total. Experiment 1 verified the effect of ad color type (green/color) on consumers’ preferences for green products. The results showed that, compared with green advertisements, the use of color ad was beneficial to promote consumers’ preferences for green products. Meanwhile, experiment 1 analyzed the mediating effect of the self-control system between ad color type (green/color) and consumers’ preferences. Experiment 2 further clarified the boundary of the main effect. The effect of ad color type (green/color) on consumers’ preferences was only valid in the context of green products. Experiment 3 explored the moderating role of green product type (egoistic/altruistic) on the main effect. The results showed that ad color type (green/color) significantly affected consumers’ preferences only when the green product type was altruistic.

## Literature review

### Green product advertising

The birth of green products stems from consumers’ environmental awareness (Tseng and Hung, 2013). More and more consumers consider the negative impact of their actions on the environment when purchasing, using or disposing of products (Carlson et al., 1993). Green products refer to products with green attributes such as energy saving, environmental protection, harmlessness, and health, or associated products, such as environmentally friendly cleaners, recycled paper, green organic food (Lin and Huang, 2012). Green consumption is an effective market strategy to address issues related to ecological hazards by providing environmentally beneficial products and services that prevent, limit, reduce or correct negative impacts on the natural environment (Rex and Baumann, 2007; Liobikienė and Bernatonienė, 2017). However, despite great efforts by companies to make the eco-labeling of green products more efficient, the majority of consumers express great concern about environmental issues, the actual market share of green products still needs to be further improved (Pancer et al., 2017).

Existing research shows that green advertising plays an important role in shaping green consumer behavior (Paek and Pan, 2004; Tsai, 2007). Kilbourne (1995) defines green advertising as green product advertisements that convey themes such as “ecology” and “human health,” and guide and shape consumers’ purchasing tendencies by showing consumers the positive characteristics of green products. Green advertising can arouse consumers’ potential green consumption awareness and effectively promote green consumption behavior by spreading green information, green knowledge, and green concepts (Yoon and Kim, 2016). Therefore, exploring the relevant factors affecting green advertising has become the focus of both business and academic circles. Elgaaied-Gambier et al. find that the type of appeal norm (descriptive/mandatory) can effectively affect the effect of green advertising, and the use of descriptive normative appeals in advertising can more effectively promote green consumption behavior. Ying and Jing find that the use of green appeal in advertisements is more likely to induce consumers’ positive advertising attitudes. Yang et al. show that abstract (concrete) appeals in advertising are more effective when green products are associated with others (self). Wang et al. find that appreciative green appeals are more effective than contemptuous green appeals in increasing consumers’ purchase intention. Septianto et al. explore the impact of visual representation on advertising persuasion and find that matching photos (illustration) with egoistic (altruistic) advertising appeals can effectively improve advertising effectiveness. Schmuck et al. find that the use of natural imagery in green advertising is more likely to evoke a positive emotional response from consumers. In general, the existing studies focus on the content and type of green advertisements, as well as the advertisement images in the visual cues, and lacks the attention and exploration of the important visual feature of ad color type (green/color). Therefore, to make up for the limitations of previous studies, this study analyzes the impact of ad color type (green vs. color) on consumers’ preferences for green products.

### Color theory (green/color)

Color is an important factor affecting consumer mood, feeling, and cognition (Reber and Schwarz, 1999; Gegenfurtner, 2003; Aslam, 2006; Wantz et al., 2015), which is used as an effective customer communication tool in marketing (Sacharow, 1975; Deng et al., 2010; Labrecque et al., 2013; Henrik and Adam 2017). Existing research shows that color can have a significant impact on consumer behavior, such as price perception (Puccinelli et al., 2013), moral judgment (Bock et al., 2013), brand personality (Labrecque and Milne, 2012), consumers perceptions, and purchase intentions (Bellizzi and Hite, 1992), advertising perceptions (Wedel and Pieters, 2015). Color can also effectively influence the subconscious level of consumers, for example, consumers do not realize that they are attracted by color when they turn their attention to the product packaging (Bagchi and Cheema, 2013). In the real market scenarios, green product advertisements usually use two colors: some enterprises use green advertisements to emphasize environmental protection attributes; others use color advertisements. Therefore, this study explores the effect of ad color type (green vs. color) on consumers’ preferences for green products.

Existing research shows that the ad color type (green/color) affects consumers’ thinking style (abstract/concrete; Stillman et al., 2020). Green as a solid color in advertising makes consumers pay more attention to product shape and outline, while color makes consumers pay more attention to the color itself (Henrik and Adam 2017; Joan and Peracchio, 1995). However, product shape outline is a basic and constant “high-level” visual feature that usually activates consumers’ abstract thinking style (Lee et al., 2014); while color is an occasional and variable “low-level” visual feature, which often activates specific thinking style of consumers (Stillman et al., 2020). Specifically, first, shape perception is less dependent on the specific environment than color, the perception of color may change with the brightness of the environment or different viewing angles, but the perception of shape tends to be stable (Arnheim, 1974). Second, compared to color, the shape can effectively convey the abstract nature of an object (Rossiter, 1982; Brockmann, 1991). For example, changing the shape of an object (car and bus) can change its meaning and nature. Conversely, changing the color of an object (blue vs. red sedan) generally does not change the essence of the object (Biederman and Ju, 1988; Mapelli and Behrmann, 1997). Therefore, shapes play a more important role in conveying the abstract nature of objects than colors (Rossiter, 1982; Brockmann, 1991). Finally, solid-color advertising highlights the outline and boundary information of the product, helps consumers focus on abstract information such as the whole of things, and reduces the contrast of the product so that the specific details of the product are less prominent and vivid (Davidoff and Rabins, 1991; Greenleaf, 2011). For example, for a solid color advertisement, consumers will pay more attention to abstract information such as the shape and function. However, colorful advertisements highlight different shades and textures of products, drawing consumers’ attention to specific details (Janiszewski, 1998; Itti and Koch, 2001). For example, for a color advertisement, consumers tend to pay attention to specific details such as the color and texture.

To sum up, green advertising, as solid-color advertising, focuses consumers’ attention on the overall shape of the product, and the shape is an abstract feature that describes the essence of the object (Arnheim, 1974), which reduces consumers’ attention to details (Mapelli and Behrmann, 1997), prompting consumers to activate a holistic, high-level, decontextualized abstract thinking style. However, color is a key factor in distinguishing details, and green product advertisements use color to focus consumers’ attention on product details (Rossiter, 1982; Brockmann, 1991), prompting consumers to activate low-level, complex, and contextualized concrete thinking style. The color theory finds that color type (green, color) can influence cognitive models (abstract, concrete). However, consumers’ cognitive models can activate relevant self-control system. Therefore, color theory establishes a theoretical connection between ad color type and self-control systems, which provides the theoretical basis for the internal mechanism of the main effect.

### Self-control system theory

In real life, we are often faced with a variety of decisions, but not all target options are attractive. The process of goal pursuit involves the individual resolving conflicts among multiple goals to ensure the achievement of multiple goals. Self-control systems theory describes how people deal with multiple conflicting goals (Fishbach et al., 2006; Koo and Fishbach, 2014). In everyday life, people often have multiple conflicting goals. For example, I want to exercise and eat healthy food to lose weight, but I cannot control myself. Self-control systems theory states that people have two dynamical systems to deal with multiple conflicting goals: the reinforcement system and the balancing system (Fishbach and Zhang, 2008). In the reinforcement system, individuals always choose alternatives that are consistent with more important goals. Individuals have a preference for behaviors by emphasizing partially accomplished goals and continuing to choose behaviors that are consistent with more important goals. In the balance system, individuals participate in different goals to maximize the benefits of all goals (Feng et al., 2016). The dynamics of reinforcement and balance generate different selection patterns that reflect the framing of specific choices as goal commitment or goal progress.

In the reinforcement system, the corresponding selection is made through the goal commitment, while in the balance system, the corresponding selection is mainly made through the goal progress. In the reinforcement system, the relationships between goals are competing with each other. Individuals will prioritize the goal that is the most important to themselves. Behaviors consistent with the goal will be interpreted as a commitment to that goal, and these goal-aligned behaviors will increase the priority of this goal over other competing goals (Feather, 1982; Locke and Latham, 1990). In the balance system, the relationship between goals is complimentary. Individuals will desire to maximize the benefits of all goals. Behavior consistent with the goals will be interpreted as the completion of the goal. To obtain all the benefits of all the goals as much as possible, the degree of goal completion will make the individual turn to the opposite goal. The goal choice is compatible.

Existing research shows that an individual’s way of thinking style (abstract, concrete) affects self-control systems. According to the theory of self-control systems, goals are seen as a cognitive structure that can be expressed as movement and progress towards some end state; and as a commitment to a fixed end state (Fishbach and Dhar, 2005). Goal progress refers to the pursuit of a previously defined goal, splitting the goal into small components, emphasizing the magnitude of the difference between the current state and goal achievement (Gollwitzer, 1999; Locke and Latham, 2002), so goal progress is a splintered detail. Therefore, it is easier for people to choose the goal progress under the specific thinking, which activates the balance system. In this condition, the individual is compatible with the target selection. Goal commitment is an inference about goal strength, that assesses the perceived value and likelihood of achievement of the goal (Feather, 1982), so goal commitment is usually regarded as a continuous variable. People are more inclined to guide behavior through goal commitment under abstract thinking style, which activates the reinforcement system. In this condition, the individual has priority in goal selection (Fishbach et al., 2006).

Green products are compatible products that can meet the dual goals of differentiation and assimilation. Different self-control system makes individuals’ different responses to the target selection, thus affecting their preferences for green products. When the reinforcement system is activated, the individual conducts behavior selection through goal commitment. He will be more inclined to make continuous efforts for a single goal, thus not prefer multi-objective compatible products, such as green products. However, when the balance system is activated, the individual conducts behavior selection through goal progress, hoping to meet all the goals to the maximum extent, so he is more likely to choose multi-objective compatible products, such as green products. To sum up, the self-control systems theory is an important internal mechanism to explain consumers’ attitudes toward green products. Therefore, based on the self-control systems theory, the current research analyzes the effect of advertising color type (green vs. color) on consumers’ preferences for green products.

## Hypothesis development

### The impact of green advertising color types on consumer preference

Advertising is an important influencing factor in consumers’ purchasing behavior (Heiser et al., 2008; Green and Peloza, 2014; Elgaaied-Gambier et al., 2018). Color is also an important part of the visual characteristics of advertising. Consumers will use advertising color as an external cue to evaluate products (Petty et al., 1983; Stillman et al., 2020). Advertising color has a great influence on consumers’ purchasing preferences (Seo and Scammon, 2017).

This study argues that the color type (green/color) of green product advertisements will activate different thinking styles of consumers, thereby affecting their self-control systems and ultimately resulting in different product preferences. Each individual has the dual psychological goals of pursuing uniqueness and a sense of belonging to the group (Brewer, 1991; Hornsey and Jetten, 2004). Individuals have both assimilation (integration into the group) and differentiation (different) needs. These are two fundamental, contradictory psychological goals that exist simultaneously in the individual. Assimilation is the oldest topic in psychology and consumer research (Asch, 1955; Burnkrant and Cousineau, 1975). People usually behave like the people around them. For example, they listen to the same music as the people around them, and they buy clothes that are popular in the season to help them better integrate into the group. Choosing the same or similar things is the most direct way to gain group identification (Berger and Heath, 2007). But people also want to be different to meet the psychological needs of differentiation. For example, when individuals want to stand out in an exchange or interview, they buy shirts with unique logos or wear some special trinkets (Tian et al., 2001). Existing research shows that consumers do not have a single need for assimilation or differentiation, and these two conflicting psychological goals often coexist (Vignoles et al., 2000; White and Dahl, 2006; Berger and Rand, 2008). Therefore, two contradictory psychological goals (assimilation, differentiation) coexisting in an individual will jointly influence their attitude toward green products.

Green products have dual functions (Olson et al., 2016; Berger, 2019; Yan et al., 2021). On the one hand, buying green products requires self-sacrifice and a willingness to give up some personal interests for the environment (Sexton and Sexton, 2014). Therefore, individuals who engage in green consumption are seen as friendlier, more cooperative, more ethical, and more selfless (Mazar and Zhong, 2010; Wang et al., 2020), and these moral attributes of consuming green products can make individuals better integrated into the group (Cottrell et al., 2007), to achieve the psychological goal of individual assimilation. On the other hand, purchasing green products as prosocial behavior requires a more complex understanding (Maloney et al., 1975) and the ability to bear higher costs (Olson et al., 2016). Therefore, individuals who engage in green consumption tend to have higher social status and higher reputation (Griskevicius et al., 2010) and can promote self-improvement through green consumption to differentiate themselves from others in the social hierarchy, thereby achieving the psychological goal of consumer differentiation. To sum up, green products have the dual functions of assimilation and differentiation.

In the context of this research, the color type (green/color) of green advertisements can activate different thinking styles of consumers and then influence consumers’ preferences for green products. Specifically, when green advertising uses green, it will activate the abstract thinking style of consumers, thereby inspiring individuals to strengthen the system. At this time, consumers are likely to pay attention to the meaning of product selection to themselves from the perspective of goal commitment, by emphasizing the pursuit of partially completed goals and the continuous selection of behaviors that are consistent with more important goals. The choice of target behaviors is single and exclusive, which conflicts with the dual attributes of green products (representing both differentiation and assimilation psychological goals), thereby reducing the consumers’ preferences for green products. When the green advertisement adopts color, it will lead to the specific thinking style of consumers and activate the balance system of the individual. At this time, consumers will tend to complete all goals to maximize the benefits and have stronger compatibility with the goals. Green products can meet the dual psychological goals of individual differentiation and assimilation at the same time, and are more in line with consumer needs under a balanced system, thereby increasing consumers’ preferences for green products.

*H1a:* Advertising color type (green/color) affects consumers’ preferences for green products. Using color ad is more likely to increase consumers’ preferences for green products than using green ad.

*H1b:* Self-control systems mediate the relationship between advertising color type (green/color) and consumers’ preferences.

### The moderating effect of product type (egoistic vs. altruistic)

According to previous studies, green products can be divided into egoistic and altruistic (Kong and Zhang, 2014; Septianto et al., 2019). Egoistic green products (oriented to personal interests) refer to the fact that the green attributes advertised in the products have a direct interest relationship with consumers themselves (Peloza et al., 2013; Green and Peloza, 2014). For example, eating more green organic vegetables are good for health. However, altruistic green products (oriented to the interests of others) refer to those whose main beneficiaries of the green attributes advertised in green products are other individuals or society as a whole (Griskevicius et al., 2010; Septianto et al., 2019). For example, the use of this green product can reduce environmental pollution and protecting nature.

This study argues that green product type (egoistic/altruistic) can effectively moderate the effect of advertising color type (green/color) on consumers’ preferences for green products. Specifically, green product types affect consumers’ processing motivation and invested cognitive resources (Anand and Sternthal, 1989), which in turn affects consumers’ self-control systems and product preferences. When the attributes of green products are related to their interests (egoistic products), because the products are closely related to their interests, consumers will have a strong motivation to process and invest more cognitive resources (Petty et al., 1983), pay attention to all relevant details of the product (Miniard et al., 1991), and are less susceptible to peripheral cues (Petty et al., 1983). At this time, regardless of whether the advertising color type is pure green or colorful, consumers tend to adopt a specific way of thinking (Yang et al., 2015), which activates the balance system. Advertising color type (green vs. color) did not affect consumers’ preferences for green products. However, when a green product is altruistic, consumers tend to think that the product is related to the welfare of others or society which has no direct connection with personal interests (Liberman and Trope, 2008). At this time, consumers are less motivated to process, invest less cognitive resources, and are more likely to be influenced by peripheral cues (e.g., the color of an advertisement; Petty et al., 1983). At this time, the type of advertising color (green/color) will activate different self-control systems by affecting consumers’ thinking styles, resulting in different green product preferences. In this condition, the use of color ad can elicit higher consumers’ preferences for green products than green ad.

*H2a:* When the green product type is egoistic, the advertising color type (green/color) does not affect consumers' preferences.

*H2b:* When the type of green product is altruistic, the use of color ad is more likely to increase consumers' preferences than the use of pure green ad.

## Research methods

### Experiment 1

Experiment 1 aims to test hypothesis 1, that the type of advertising color (green/color) can effectively influence consumers’ preferences for green products. Using color ad is more likely to improve consumers’ preferences than using green ad.

#### Participants and design

Study 1 used a one-factor (product color: green vs. color) between-participant design. Based on the calculation method in Cohen (2013) and the effect size (effect size *f* = 0.25; power = 0.80) of related studies (Petty et al., 1983), G*Power 3.1 software was used to calculate the sample size which was more than 128 people. Therefore, experiment 1 recruited 150 participants in a school with a reward of 10 yuan to complete a series of survey activities on green battery product. Participants were randomly assigned to two groups (pure green ads/color ads), and the final sample size was (*N* = 137, age 18 ~ 35, *M*_age_ = 23.39, SD = 3.06, 45.98% female), and the sample in each group was (*n*_green_ = 70, *n*_color_ = 67).

#### Procedure and stimuli

To ensure the effectiveness of the green product operation, the researchers randomly recruited 50 participants (*N* = 50, ages 20 to 38, *M*_age_ = 25.86, SD = 4.53, 54.00% female) online for a pre-test. The researchers asked participants to read the relevant information of the green battery: “*Green battery refers to a type of high-performance, a non-polluting battery that has been put into use or is being developed and developed. This new type of alkaline battery has high energy, is beneficial to environmental protection and long life and other advantages*,” and the corresponding battery picture. Then the participants reported whether the battery was a green product (saving energy, protecting the environment, and being renewable). The results showed that all participants considered the battery to be a green product, which ensured the effectiveness of the green product in Experiment 1.

Then, in the main experiment, the researchers introduced information about the green battery to the participants, expressing their desire to solicit consumers’ opinions on the new product. According to the group of participants (green, color), the researchers gave green battery advertisement pictures in corresponding colors. Afterward, participants were invited to fill out a survey report, including the degree of preference for the target green battery (7-point scale, 1 = “very dislike,” 7 = “very much like”; Lee and Pillai, 2013), to assess the consumer’s self-control system (Feng et al., 2016; 1–7 points scale, 1 point = only focus on maximizing all benefits when choosing, 7 = only focus on the importance of the option to yourself when choosing). To exclude other possible mediating effects, participants also reported color appropriateness (Lim et al., 2020), cognitive fluency (Novemsky et al., 2007), emotional state (Pancer et al., 2017; see Table 1). Participants also reported opinions on the product, familiarity with the product, previous buying experience, personal interests, and other confounding items. Finally, the researchers asked participants to recall the color of product advertisements in the experiment, their preferences for advertising colors (Seo and Scammon, 2017), whether they thought the target product was green, whether their preferences for the green battery depended on past experiences, and guess the purpose of this investigation.

**Table 1:** Study 1: scale.

Product preference	Please report the preference for the target product (7-point scale, 1 = “very dislike,” 7 = “very much like”)	Lee and Pillai (2013)
Self-control system	Please assess the self-control system when you choose the green product (1–7 points scale, 1 point = only focus on maximizing all benefits when choosing, 7 = only focus on the importance of the option to yourself when choosing)	Feng et al. (2016)
Color appropriateness	The color of the advertisement is appropriate for the target product (7-point scale, 1 = “totally disagree,” 7 = “totally agree”)	Lim et al. (2020)
Cognitive fluency	Please assess the complexity to read the advertisement (7-point scale, 1 = hard, 7 = easy)	Novemsky et al. (2007)
Emotional state	Please report your emotional state (7-point scale, 1 = unhappy, 7 = happy)	Pancer et al. (2017)
Manipulation check	How much do you like the color of the AD (7-point scale, 1 = very dislike, 7 = very like)	Seo and Scammon (2017)
	Please recall the color of the advertisement	
	Do your think the target product is a green product?	
	Do your preferences for the green battery depend on past experiences?	
	Please guess the purpose of this investigation	

#### Results and discussion

##### Manipulation checks

Six participants reported the wrong color, 7 participants’ preferences for products relied on the past shopping experiences, all participants considered the target product to be a green product, and no participant guessed this survey’s real purpose. There was no significant difference in the color preference of the two groups (*M*_green_ = 4.66, SD = 0.74, *M*_color_ = 4.60, SD = 0.63, *t* (135) = 0.51, *p* < 0.001, *d* = 0.09). The operation effectively affected most of the participants.

##### Green product preference

The results found that there was a significant difference in the preference of the two groups for the green product. The participants in the color advertising group have a higher product preference than the pure green advertising group (*M*_green_ = 4.11, SD = 0.69, *M*_color_ = 4.87, SD = 0.69, *t* (135) = 6.34, *p* < 0.001, *d* = 1.10), the results supported hypothesis 1.

##### Self-control system

The results showed that the self-control system of the two groups was significantly different. The participants in the color group were more likely to activate the balance system than the green group (*M*_green_ = 3.81, SD = 0.73, *M*_color_ = 4.37, SD = 0.62, *t* (135) = 4.82, *p* < 0.001, *d* = 0.83).

##### Single-mediation analysis

**Figure 1:**
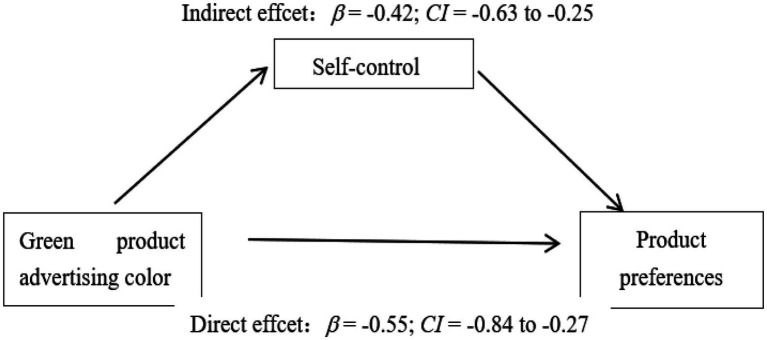
The Mediating effect of Self-control System.

To further verify the mediating effect of the self-control system between the advertising color of green products and consumers’ preferences, experiment 1 analyzed the mediation effect of the self-control system through bootstrapping (using PROCESS Model 4). The results showed that the self-control system mediated the effect of advertising color type on consumers’ preferences (95% confidence interval *β* = −0.42, CI = −0.63 to-0.25; more details see Figure 1).

##### Multi-mediation analysis

Using SPSS Macro software proposed by Preacher and Hayes (2008), with advertising color type as the independent variable, consumers’ preferences as the dependent variable, self-control system, cognitive fluency, color suitability, and emotion as the mediator variables for multiple mediation analysis.

The results showed that advertising color type (green, color) significantly affected the self-control system (*β* = −0.56, *t* = −4.82, *p* < 0.001), but not cognitive fluency (*β* = 0.25, *t* = 1.42, *p* = 0.159), color suitability (*β* = 0.06, *t* = 0.41, *p* = 0.684), and mood (*β* = 0.15, *t* = 1.17, *p* = 0.245). Controlling for other mediators, the self-control system was able to significantly mediate the relationship between advertising color type (green, color) and consumers’ preferences (*β* = −0.42, *p* < 0.001, CI95: low = −0.62; high = −0.24). However, cognitive fluency (*β* = 0.006, *p* < 0.001, CI95: low = −0.01; high = 0.05), color suitability (*β* = −0.001, *p* < 0.001, CI95: low = −0.03; high = 0.01) and mood (*β* = 0.003, *p* < 0.001, CI95: low = −0.01; high = 0.04) did not have this indirect effect (Table 2).

**Table 2:** Study 1: result.

	Green *M* + SD	Color *M* + SD	*t*
Color preference	4.66, 0.74	4.60, 0.63	*t* = 0.51
Green product Preference	4.11, 0.69	4.87, 0.69	*t* = 6.34
Self-control System	3.81, 0.73	4.37, 0.62	*t* = 4.82
Model Path Estimates			
Indirect effect (with Bootstrap 95% Confidence Interval and Standard Errors)			
	**Effect**	**LL95% CI**	**UL95% CI**
Consumer preference	−0.42	−0.62	−0.24
Cognitive fluency	0.006	−0.01	0.05
Color suitability	−0.001	−0.03	0.01
Mood	0.003	−0.01	0.04

##### Discussion

Experiment 1 discovered that advertising color type (green/color) affected consumers’ preferences for green products. Using color ad is more likely to increase consumers’ preferences for green products than using green ad. At the same time, experiment 1 verified the mediating role of the self-control system between ad color types and consumers’ preferences, revealing the theoretical logic of the main effect. Experiment 2 introduced the product attribute (green product/common product) as a moderator, which further explored boundary conditions for the main effect.

### Experiment 2

Experiment 2 aims to further clarify the boundaries of the main effect, that is, the effect of advertising color (green/color) on consumers’ preferences is only significant in the context of green products.

#### Subject selection

Study 2 employed a 2 (ad color: green vs. color) × 2(product type: green product vs. normal product) between-participants design. Based on the calculation method in (Cohen, 2013) and the effect size (effect size *f* = 0.25; power = 0.80) of related studies (Leenaars et al., 2016; Miao et al., 2021), G*Power 3.1 software calculated the sample size which was more than 179 people. Therefore, Experiment 2 recruited 210 participants in a school with a reward of 10 yuan to complete a series of survey. Participants were randomly assigned into a 2 (pure green/color) * 2 (green product/normal product) experimental design. The final sample size was 192 (ages 18–34, *M*_age_ = 23.25, SD = 3.39, 52.08% female). The sample size of each group was (*n*_green products – green_ = 47, *n*_green products – color_ = 48, *n*_normal products – green_ = 51, *n*_normal products – color_ = 46).

#### Procedure and stimuli

To ensure the effectiveness of the product attribute (green/normal) in experiment 2, the researchers recruited 85 participants (ages 20–35, *M*_age_ = 24.62, SD = 3.56, 49.41% female) online for a pre-test. Participants were randomly divided into two groups (green product group/normal product group), and corresponding product information was provided for each group. The information obtained by the green product group was (*this is a natural laundry detergent extracted from plant soap-based raw materials, without phosphorus, fluorescent agents, whitening agents, and other harmful ingredients, and is very safe for skin health*); the normal product group obtained the information was (*this is a laundry detergent that dissolves quickly, has strong detergency, and has the excellent suspending ability, making clothes as white as new*). Afterward, participants were asked to rate the product type of laundry detergent (7-point scale, 1 = this product is a normal product, 7 = this product is a green product). The results showed that participants in the green product group rated the product’s green attributes significantly higher than the normal product group (*M*_normal_ = 1.74, SD = 0.63, *M*_green_ = 5.86, SD = 0.68, *t*(83) = 29.15, *p* < 0.001, *d* = 6.29), which ensured the validity of the product attribute of Experiment 2.

Then, in the main experiment, the researchers introduced information about the laundry detergent to the participants, hoping to solicit consumers’ opinions on the laundry detergent. The researchers provided each group of participants (green, color) with the advertisement of different color (green, color). For consumers in the green product group, the researcher introduced relevant information about the green product, and for consumers in the normal product group, the researcher introduced relevant information about the normal product (see the pre-test for details). Afterward, participants were asked to fill out a survey report that included the preference for the target laundry detergent, an assessment of the self-control system, and some other confounding items. Finally, participants were asked to recall the color of the advertisement in the experiment, the degree of preference for the color of the advertisement, whether the preference for green products depended on past experiences, and guessed the purpose of this investigation (Table 3).

**Table 3:** Study 2: scale.

Product preference	Please report the preference for the target product (7-point scale, 1 = “very dislike,” 7 = “very much like”)	Lee and Pillai (2013)
Self-control system	Please assess the self-control system when you choose the green product (1–7 points scale, 1 point = only focus on maximizing all benefits when choosing, 7 = only focus on the importance of the option to yourself when choosing)Feng et al., (2016)	
Manipulation check	How much do you like the color of the AD (7-point scale, 1 = very dislike, 7 = very like)	Seo and Scammon (2017)
	Please recall the color of the advertisement	
	Do your think the target product is a green product?	
	Do your preferences for the green battery depend on past experiences?	
	Please guess the purpose of this investigation	

#### Results and discussion

##### Manipulation checks

Eight participants reported the wrong color, 10 participants’ preferences for target products relied on the past shopping experiences, and none of the participants guessed the true purpose of this survey. There was no significant difference in preference for advertising color between the two groups (*M*_color_ = 4.43, SD = 0.77, *M*_green_ = 4.52, SD = 0.94, *t* (190) = 0.76, *p* = 0.447, *d* = 0.10). Participants in the green product group rated product attributes significantly higher than those in the normal product group (*M*_green product group_ = 5.29, SD = 0.67, *M*_normal product group_ = 2.49, SD = 0.83, *t* (190) = 25.73, *p* < 0.001, *d* = 3.71), the operation effectively affected the majority of participants.

##### Product preference

The results showed that the interaction of advertising color type (color/green) and product attributes (green product/normal product) significantly affected consumers’ preferences (*F* = 4.36, *p* < 0.05). There was no significant difference in product preference between the two groups in the common product context (*M*_green_ = 4.53, SD = 1.35, *M*_color_ = 4.50, SD = 1.17, *t* (95) = 0.11, *p* = 0.909, *d* = 0.02). However, in the context of green product, there was a significant difference in the product preferences of the two groups. The color group reported a higher preference than the green group (*M*_green_ = 4.36, SD = 0.74, *M*_color_ = 4.98, SD = 0.91, *t* (93) = 3.63, *p* < 0.001, *d* = 0.75).

##### Self-control system

The results showed that the interaction of product advertising color type (color/green) and product attributes (green product/normal product) did not affect consumers’ self-control system (*F* = 1.07, *p* = 0.303). In the general product situation, there were significant differences in the self-control system of the two groups (*M*_green_ = 3.73, SD = 0.67, *M*_color_ = 4.37, SD = 0.80, *t* (95) = 4.33, *p* < 0.001, *d* = 0.87). In the context of green product, there were also significant differences in the self-control system of the two groups (*M*_green_ = 3.85, SD = 0.78, *M*_color_ = 4.27, SD = 0.76, *t*(93) = 2.65, *p* < 0.05, *d* = 0.55).

##### Mediating moderating effect analysis

**Figure 2:**
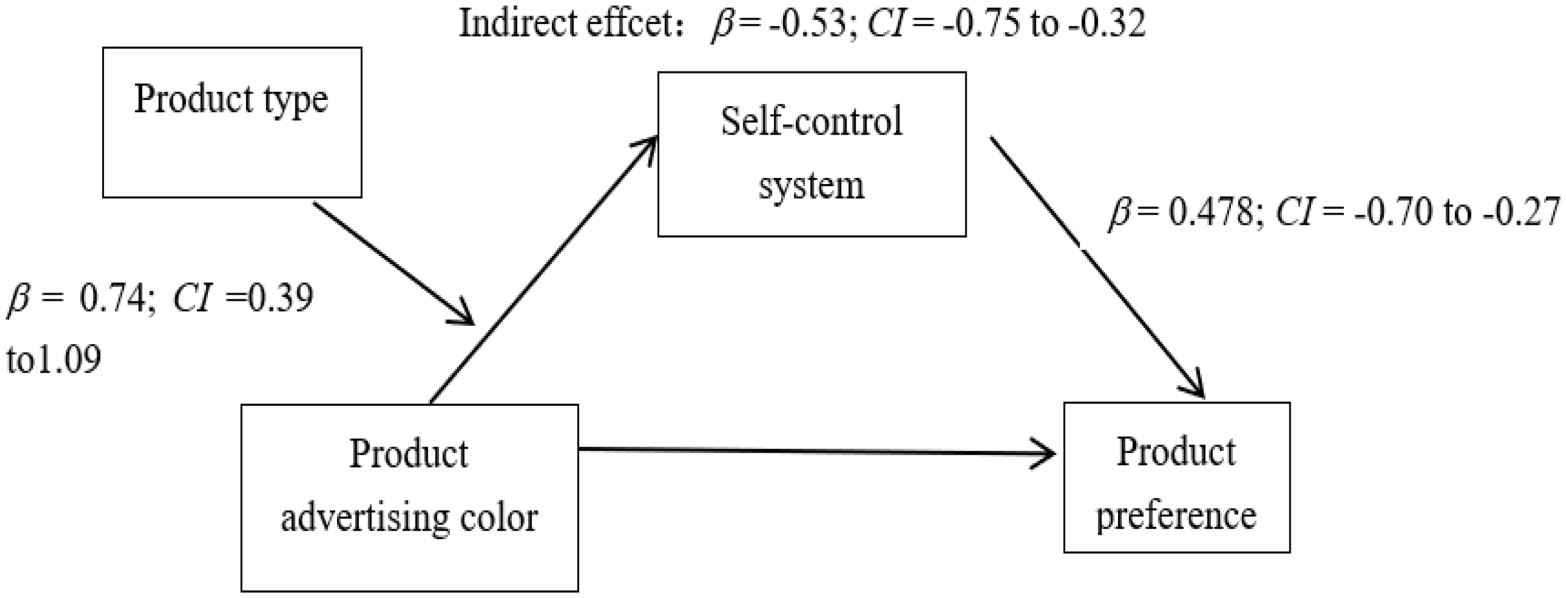
The Moderating Role of Product Type.

In this study, ad color (green/color) was used as the independent variable, consumers’ preferences were the dependent variable, self-control system was the mediating variable, and product attributes (green product/common product) were the moderating variable. Using bootstrapping (PROCESS Model 14) to analyze the moderating effect of product attributes. The results showed that product advertisement color significantly affected the self-control system (95% confidence interval *β* = −0.53; CI = −0.75 to –0.32), while the interaction between the self-control system and product type significantly affected consumers’ preferences (95% confidence interval *β* = 0.74; CI = 0.39–1.09). Product advertising color was able to influence consumers’ preferences through a self-control system when the product was green (conditional indirect effect, 95% confidence interval *β* = −0.47; CI = −0.70 to –0.27). When the product is a normal product, product advertising color did not influence consumers’ preferences through a self-control system (conditional indirect effect, 95% confidence interval *β* = −0.08; CI = −0.28 to 0.12), as shown in (Figure 2; Table 4).

**Table 4:** Study 2: result.

Manipulation checks	*M* + SD	*M* + SD	*t*
Color preference (green, color)	4.52, 0.94	4.43, 0.77	*t*_=_ 0.76
Color preference (green, normal)	5.29, 0.67	2.49, 0.83	*t*_=_ 25.73
Product preference	Green *M* + SD	Color *M* + SD	T
Common products	4.53, 1.35	4.50, 1.17	*t*_=_ 0.11
Green products	4.36, 0.74	4.98, 0.91	*t* = 3.63
Self-control system	Green *M* + SD	Color *M* + SD	T
Common products	3.73, 0.67	4.37, 0.80	*t*_=_ 4.33
Green products	3.85, 0.78	4.27, 0.76	*t* = 2.65
Model path estimates indirect effect (with Bootstrap 95% Confidence Interval and Standard Errors)			
	**Effect**	**LL95% CI**	**UL95% CI**
Color—self-control system	−0.53	−0.75	−0.32
Self-control system × product type–preference	0.74	0.39	1.09
Color–Preference–Self-control system (green)	−0.47	−0.70	−0.27
Color–Preference–Self-control system (normal) –0.08	−0.28	0.12	

##### Discussion

Experiment 2 further verified the theoretical logic of the main effect by manipulating product attributes (green product/normal product) which clarified the boundary of the main effect. The influence of advertising color type (green, color) on consumers’ preferences was only effective in the context of green product. To reveal the boundary conditions of the main effect, this study employed Experiment 3 to analyze the moderating effect of product attributes (egoistic/altruistic) on the relationship between green product ad color types and consumers’ preferences.

### Experiment 3

Experiment 3 explores the moderating effect of green product type (egoistic/altruistic) on the relationship between ad color type (green/color) and consumers’ preferences, which supports hypothesis 2.

#### Design and participants

Based on the calculation method in (Cohen, 2013) and the effect size (effect size *f* = 0.25; power = 0.80), G*Power 3.1 software calculated the planned sample size of more than 179 people. Therefore, Experiment 3 recruited 210 participants in a school with a reward of 10 yuan to complete a series of survey. Participants were randomly assigned to an experimental design of two ad colors (green vs. color) * 2 product types (egoistic/altruistic). Finally, the total sample size was *N* = 188 (ages 18–37, *M*_age_ = 24.61, SD = 4.19, 51.60% female), the sample size of each group was (*n*_green, egoistic_ = 48, *n*_green, altruistic_ = 47, *n*_color, egoistic_ = 47, *n*_color, altruistic_ = 46).

#### Procedure and stimuli

To ensure the effectiveness of the green product type (egoistic/altruistic), the researchers randomly recruited 82 participants (ages 18–36, *M*_age_ = 24.57, SD = 4.11, 47.56% female) online for a pre-test. The participants were randomly divided into two groups (egoistic/altruistic), and the corresponding green product information was presented to each group. The product information obtained by the altruistic group was “*This is an energy-saving car, and the pollution generated during the manufacturing process is less. With fewer emissions, lower emissions, and reduced air pollution, the long-term use of the car will have a positive impact on the environment*.” The product information obtained by the egoistic group was: “*This is an energy-efficient car that meets your interests. It is cheaper than a conventional car, and it consumes less fuel, further saving you money on fuel purchases. You can also get government environmental subsidies for making purchases*.” Afterward, participants were asked to evaluate whether the target product was considered as a green product, report the type of the green product (7-point scale, 1 = the car benefits the consumer’s interests, 7 = the car benefits the interests of other members of society). The results showed that the majority of participants believed that the target product was green. The egoistic group reported significantly lower scores on product type than the altruistic group (*M*_egoistic group_ = 2.76, SD = 1.08, *M*_altruistic group_ = 5.38, SD = 1.84, *t* (80) = 12.22, *p* < 0.001, *d* = 1.74), which ensured the validity of the manipulation in Experiment 2.

In the main experiment, the researcher informed that the activity was a market survey of a new product. Afterward, participants were presented with corresponding advertisement images and product information. In the operation of advertisement color, participants in different groups (green/color) get advertisement pictures of corresponding colors. In the operation of product type, the participants in the egoistic group received egoistic product information; the participants in the altruistic group gained altruistic product information. Afterward, participants were asked to fill out a survey report that included preferences for the target car product, an assessment of the self-control system, and some other items. Finally, participants were asked to rate whether the car was a green product, the type of product (egoistic, altruistic), recall the color of the advertisement in the experiment, the degree of preference for the color of the advertisement, whether the preference for the product depended on past experiences, and guessed the purpose of this investigation (Table 5).

**Table 5:** Study 3: scale.

Product preference	Please report the preference for the target product (7-point scale, 1 = “very dislike,” 7 = “very much like”)	Lee and Pillai (2013)
Self-control system	Please assess the self-control system when you choose the green product (1–7 points scale, 1 point = only focus on maximizing all benefits when choosing, 7 = only focus on the importance of the option to yourself when choosing) Feng et al., (2016)	
Manipulation check	How much do you like the color of the AD (7-point scale, 1 = very dislike, 7 = very like)	Seo and Scammon (2017)
	Please recall the color of the advertisement	
	Do your think the target product is a green product?	
	Do your preferences for the green battery depend on past experiences?	
	Please guess the purpose of this investigation	

#### Results and discussion

##### Manipulation checks

Ten participants reported wrong ad color, 12 participants’ preferences for the product relied on the past shopping experiences, and none of the participants guessed the true purpose of this survey. There was no significant difference in preference for advertising color between the two groups (*M*_green_ = 4.44, SD = 0.94, *M*_color_ = 4.48, SD = 1.13, *t* (186) = 0.28, *p* = 0.783, *d* = 0.04). Participants in the egoistic group reported lower results on the product type than the altruistic group (*M*_egoistic group_ = 2.56, SD = 0.77, *M*_altruistic group_ = 5.02, SD = 0.81, *t* (186) = 21.44, *p* < 0.001, *d* = 3.11), the manipulation effectively affected most of the participants.

##### Self-control system

The results showed that the interaction between ad color and product type significantly affected participants’ self-control system (*F* = 5.65, *p* < 0.05). In the context of egoistic products, there was no significant differences between the two groups (*M*_green_ = 3.71, SD = 0.62, *M*_color_ = 3.72, SD = 1.08, *t* (93) = 0.08, *p* = 0.933, *d* = 0.01). However, in the context of altruistic products, there was a significant difference in the self-control system of the two groups. The color group reported higher results than the green group (*M*_green_ = 3.64, SD = 1.09, *M*_color_ = 4.28, SD = 0.75, *t* (91) = 3.31, *p* < 0.001, *d* = 0.68).

##### Product preference

The results showed that the interaction of ad color and product type significantly affected participants’ preferences (*F* = 5.93, *p* < 0.05). In the case of egoistic product, product preferences did not differ between the two groups (*M*_green_ = 4.08, SD = 0.82, *M*_color_ = 4.15, SD = 1.08, *t*(93) = 0.33, *p* = 0.740, *d* = 0.07). However, in the case of altruistic products, there was a significant difference between the two groups. The color group reported higher preferences than the green group (*M*_green_ = 4.09, SD = 1.06, *M*_color_ = 4.85, SD = 0.94, *t* (91) = 3.67, *p* < 0.001, *d* = 0.76). The results supported hypothesis 2.

##### Analysis of mediating moderating effect

**Figure 3:**
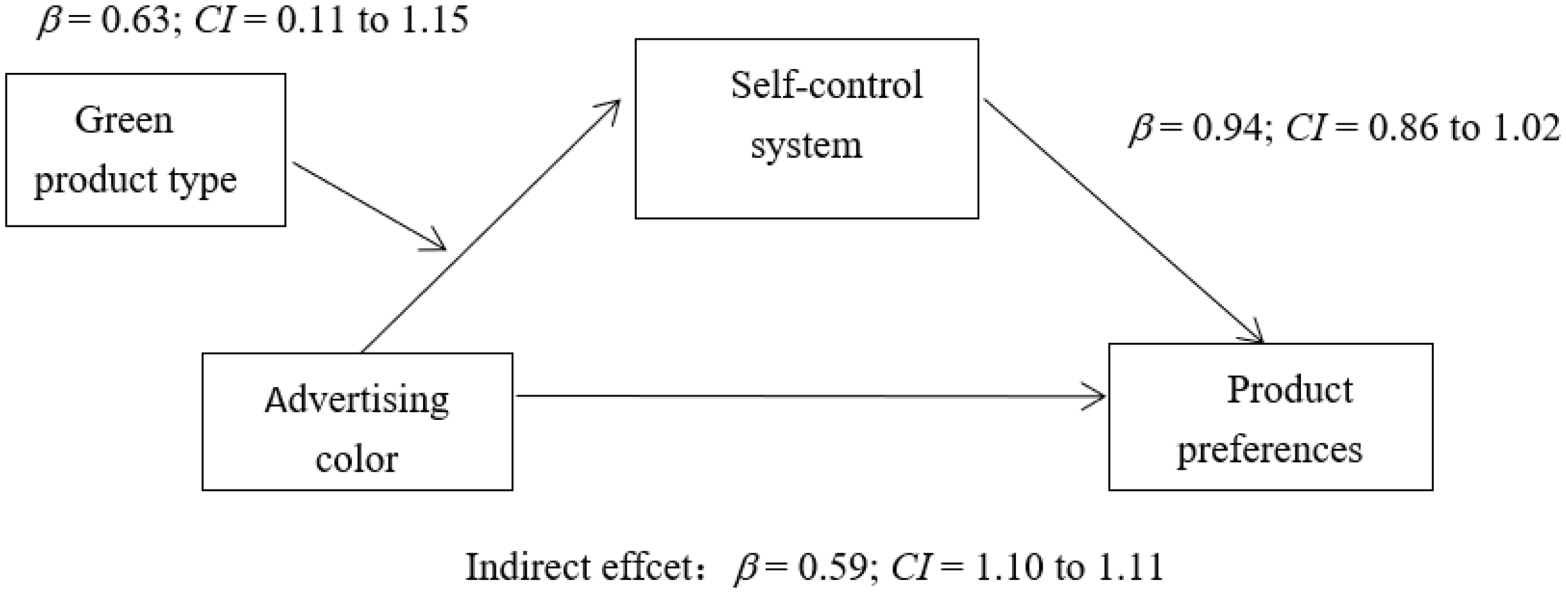
The Moderating Role of Green Product Type.

Since the product type (moderating variable) moderated the relationship between green product ad color and consumers’ preferences by affecting the self-control system (mediating variable), this study used Bootstrapping (PROCESS Model 7) to analyze the moderating effect of product type. The results showed that the interaction between the ad color (color/green) and the product type could significantly affect the individual’s self-control system (95% confidence interval *β* = 0.63, CI = 0.11–1.15). Self-control system also effectively influence individuals’ preferences for green products (95% confidence interval *β* = 0.94, *CI* = 0.86–1.02). When the green product was altruistic, ad color significantly influenced product preference through the self-control system (conditional indirect effect, 95% confidence interval *β* = −0.61; CI = −0.99 to –0.25). When the green product was egoistic, the indirect effect of green product ad color on consumers’ preferences was not significant (conditional indirect effect, 95% confidence interval *β* = −0.01; CI = –0.34–0.32). In conclusion, product type effectively moderated the relationship between green product ad color and consumers’ preferences through the self-control system (95% confidence interval *β* = 0.59, CI = 0.10–1.11), as shown in Figure 3.

##### Discussion

Experiment 3 found that green product type (egoistic, altruistic) effectively moderated the effect of ad color (green, color) on consumers’ preferences for green product, which supported hypothesis 3. When the green product type was egoistic, the ad color type (green/color) did not affect consumers’ preferences; when the green product type was altruistic, color ad was more likely to increase consumers’ preferences than green ad.

## Conclusion

This study demonstrated the influence of green product ad color (green/color) on consumers’ preferences through three experiments which constructed a complete internal mechanism and boundary conditions. Experiment 1 discovered the effect of ad color type (green/color) on consumers’ preference for green products. Color ad improved consumers’ preferences more than pure green ad. Meanwhile, experiment 1 analyzed the mediating role of the self-control system and verified the causal chain model from the ad color type of green product to the self-control system to consumers’ preferences. Experiment 2 further clarified the boundaries of the main effect, confirming that the impact of ad color type (green vs. color) on consumers’ preferences was only significant in the context of green product. Experiment 3 explored the moderating role of product type on the main effect. The results showed that when the green product type was egoistic, the ad color type (green/color) did not affect consumers’ preferences for green products; when the green product type was altruistic, color ad was more likely to increase consumers’ preferences for green products than green ad.

## Discussion

### Theoretical contributions

The theoretical contributions of this research are mainly reflected in the following aspects:

First, this study enriches the research on the color of green product advertisements. As an important carrier of green information, knowledge, and ideas, green product advertising can effectively promote green consumption behavior (Yoon and Kim, 2016; Wang et al., 2017; Elgaaied-Gambier et al., 2018). While previous studies have explored the impact of green advertising on attractiveness (Elgaaied-Gambier et al., 2018) and visual expression (Schmuck et al., 2018; Septianto et al., 2019). This study enriches related research on green product advertising by exploring the effectiveness of ad color type (green/color). Prior research has extensively explored the drivers of purchasing green products or consumer attitudes towards green products (Carrington et al., 2014; Haws et al., 2014; Bodur et al., 2015), but few articles explore the influence of ad color type (green/color) on consumers’ preferences. Consumers use advertising color as an external cue to evaluate products (Petty et al., 1983), make affective responses (e.g., preferences, feelings) and behavioral responses (e.g., shopping intentions, product choices; Babin et al., 2003; Labrecque and Milne, 2012). Therefore, ad color will have an impact on consumers’ purchasing preferences. Among the few studies on the effect of product ad color, they mainly focus on two distinct colors, black and white (Lee et al., 2014) or green and red (Bagchi and Cheema, 2013). These studies have found that color affects consumers’ information processing, prompting consumers to invest in different cognitive resources (Joan and Peracchio, 1995). However, little literature has explored whether two different color types, color or pure, which also affect consumers’ information processing and attitudes toward products. Because of the particularity of green products, some companies often use pure green ad color in their advertisements to show their environmental protection attributes. At the same time, a large number of color advertisements for green products also exist in the market. Therefore, this study explores the influence of ad color type (color, green) on consumers’ preferences for green products This study is the first to link the ad color of green products with product preferences. The findings confirm that the use of color ad for green products can elicit higher consumers’ preferences than pure green ad, extending the application value of color theory. Secondly, this study tests the mediating mechanism of the effect of green product ad color type on consumers’ preferences based on self-control systems theory. Although previous research has pointed out that color affects consumers’ thinking style (abstract/concrete; Stillman et al., 2020). No research has directly proposed and tested that ad color type activates different self-control systems and influenced subsequent preferences for green product. Green products have dual attributes (assimilation/differentiation; Berger, 2019; Yan et al., 2021). This study matches the dual attributes of green products with different self-control systems and establishes a relationship between ad color type and consumers’ preferences. Therefore, another contribution of this study is to identify the theoretical link between green product ad color type and consumers’ preferences through self-control systems which explains the relationship between the implicit psychological systems and the dual attributes of green products. This study expands the research on self-control systems in the field of consumer behavior and provides a new theoretical perspective for subsequent research.

Finally, this study also explores the moderating role of product type, establishing clear boundary conditions for the main effect. This study finds that product type can moderate the relationship between ad color type and consumers’ preferences. Therefore, this study enriches the theoretical research on ad color in different contexts When the green product type is altruistic, using color ad is more likely to improve consumers’ preferences for green products than pure green ad. This research embeds product types into the main research framework of green products and builds a clear boundary condition in the theoretical and application fields.

### Management implications

The conclusions of this study have practical implications for the ad color of green products. Green product companies often choose the ad color to convey different product information to the market. This study focuses on examining the market effect of green product ad color and provides practical suggestions for companies on how to design ad color effectively by exploring the impact of ad color type (pure green/color) on consumers’ preferences. First, color is an important external cue to influence consumers’ decision-making. The ad color of green products effectively affects consumers’ self-control system, resulting in different product preferences. Green products should use color advertisements instead of pure green advertisements so that the dual functions of green products are consistent with the balance system of consumers. The results of this study can provide practical suggestions to green product marketing. For example, when designing green product advertisements, companies should not use pure green to highlight the green environmental protection attributes of products. Some other colors can be added to enhance consumers’ specific perception of green products. Secondly, there are certain boundary conditions for the influence of the ad color on consumers’ preferences for green products. When the green product type is self-interested, the ad color type (pure green/color) will not affect consumers’ preferences. When the product type is altruistic, using color ad is more likely to increase consumers’ preferences for green products than pure green ad.

### Future research

This study is the first to explain the effect of green product ad color type on consumers’ preferences from the perspective of self-control system theory. Future research can further explore whether other potential mechanisms mediate the effect of green product ad color on consumers’ preferences. In addition, the sample of this study is mainly students, so the sample has certain limitations. Future research should consider further extending the sample to a wider group of consumers. Second, although the results of this study are statistically significant, consumers’ product preferences are tested through a certain experimental design, but consumers’ green product preferences in real life are not explored. Therefore, future research can further explore whether the main effect in the experimental design is also significant in the realistic conditions. Finally, the current study only explored the influence of the simple moderator variable of product type on the main effect. There may exist other possible moderator variables, such as product price (high price/low price), future research can explore more boundary conditions for the main effects.

## Data availability statement

The original contributions presented in the study are included in the article/supplementary material, further inquiries can be directed to the corresponding author.

## Ethics statement

The studies involving human participants were reviewed and approved by ethics committee of Center for Psychological Science and Health, China University of Geosciences. The patients/participants provided their written informed consent to participate in this study.

## Author contributions

FW was responsible for the logical reasoning of the research topic. ZY and SX were responsible for experimental materials and data. LC was responsible for collecting literature. All authors contributed to the article and approved the submitted version.

## Funding

The authors acknowledge financial support from the National Nature Science Foundation of China (grant nos. 72172107 and 71532011).

## Conflict of interest

The authors declare that the research was conducted in the absence of any commercial or financial relationships that could be construed as a potential conflict of interest.

## Publisher’s note

All claims expressed in this article are solely those of the authors and do not necessarily represent those of their affiliated organizations, or those of the publisher, the editors and the reviewers. Any product that may be evaluated in this article, or claim that may be made by its manufacturer, is not guaranteed or endorsed by the publisher.
